# Production and Partial Characterization of *α*-Amylase Enzyme from Marine Actinomycetes

**DOI:** 10.1155/2021/5289848

**Published:** 2021-12-07

**Authors:** Mohamed H. Al-Agamy, Mohammad R. Alhuzani, Mahmoud S. Kelany, Moaz M. Hamed

**Affiliations:** ^1^Department of Pharmaceutics, College of Pharmacy, King Saud University, 11451 Riyadh 2457, Saudi Arabia; ^2^Microbiology Unit, College of Pharmacy Research Center, King Saud University, 11451 Riyadh 2457, Saudi Arabia; ^3^Department of Microbiology and Immunology, Faculty of Pharmacy, Al-Azhar University, Cairo, Egypt; ^4^Marine Microbiology Department, National Institute of Oceanography and Fisheries (NIOF), Egypt

## Abstract

Amylase producing actinobacteria were isolated and characterized from terrestrial environment. There are a limited number of reports investigating the marine environment; hence, in the present study, four marine enzymes were tested for their amylase production ability. On starch agar plates, the *Streptomyces rochei* strain showed a higher hydrolytic zone (24 mm) than the other isolates. Growth under optimized culture conditions using Plackett-Burman's experimental design led to a 1.7, 9.8, 7.7, and 3.12-fold increase for the isolates *S. griseorubens*, *S. rochei*, *S. parvus*, and *Streptomyces* sp., respectively, in the specific activity measurement. When applying the Box-Behnken design on *S. rochei* using the most significant parameters (starch, K_2_HPO_4_, pH, and temperature), there was a 12.22-fold increase in the specific activity measurement 7.37 U/mg. The *α*-amylase was partially purified, and its molecular weight was determined using sodium dodecyl sulfate-polyacrylamide gel electrophoresis. *α*-Amylase was particularly active at pH 6 and 65°C. The purified enzyme was most active at 65°C and pH 6, thermal stability of 70°C for 40 min, and salt concentration of 1 M with Km and Vmax of 6.58 mg/ml and 21.93 *μ*mol/ml/min, respectively. The *α*-amylase was improved by adding Cu^+2^, Zn^+2^, and Fe^+2^ (152.21%, 207.24%, and 111.89%). Increased production of *α*-amylase enzyme by *S. rochei* KR108310 leads to production of significant industrial products.

## 1. Introduction

Marine habitats are regarded as a potential resource for bioactive metabolites of enormous and unparalleled industrial value. About 20,000 natural products, including nine licensed medicines and 12 under clinical trials, have been recorded from marine sources [1]. Extensive marine secondary metabolite varieties have been used for the production of medicinal products. Microorganisms function as a complement to drug development, as they are an excellent source for synthesizing various useful enzymes. Recently, marine microbial enzymes have been found to be rapid, cost-effective to produce, and environmentally sound [2]. However, in extreme circumstances, such as under alkaline and high temperature conditions, most enzymes are more frequently unstable in industrial processes. Therefore, researchers are interested in exploring marine microbes that tolerate extreme environmental conditions as a valuable natural resource of new products, including different enzymes [3]. Actinomycetes are Gram-positive and produce various commercial enzymes [4]. Among the Actinomyces isolates, *Streptomyces* species are the most essential industrially useful organism because of their ability to produce multiple enzymes [5]. Actinomycetes contain various enzymes, including protease, amylase, lipase, pectinase, cellulase, xylanase, glutaminase, and asparaginase [6]. *Streptomyces* species produce various enzymes that are nutritionally essential for these Gram-positive bacteria, including *S. griseus*, *S. clavuligerus*, *S. thermoviolaceus*, *S. rimouses*, and *S. thermovulgaris*. Additionally, *Streptomyces* species release some extra cell enzymes [7–9], such as amylase enzymes, which are considered a potential product for many fields, including manufacturing, clinical, medicinal, analytical chemistry, ethanol production, textile, food, brewing, and distillation industries. The manufacturing industries include the production of starch and syrup [10] with divided associations to glucose, maltotriose, and maltose formation using chemical methods [11]. Amylases are commonly used as an agent to treat fruit, such as bananas, mangoes, citrus fruits, and papayas, and wash pots used in the fermentation of foodstuffs and the paper and textile industries. These enzymes come from different sources, such as actinomycetes and fungi. The microbial source is favored over other sources like animals and plants because they have been generally favored for their easier isolation in high amounts, low-cost production in a short time, and stability at various extreme conditions, and their compounds are also more controllable and less harmful [11–13]. Also, *α*-amylases can be used in scarification of starch, textiles, food, brewing, and distillation industries [14, 15].

The production rate of *α*-amylase enzymes from isolates was investigated using horticulture methods, assessing pH and element requirements, incubation time, and incubation temperature. However, there are limited studies on the production of *α*-amylase from marine *Streptomyces* spp. Therefore, this study is aimed at investigating the production of *α*-amylase from different marine actinomycetes isolates belonging to the genus *Streptomyces*, the enhancement of *α*-amylase production, and the characterization of this enzyme for various industrial processes. This manuscript has been submitted as a preprint to the Research Square site [16].

## 2. Materials and Methods

### 2.1. Chemicals and Nutrient Media

All chemicals and cultivation media were obtained from the Al-Gomhoria Chemicals Company, Egypt. All laboratory tests were conducted using sterilized seawater (collected from the study area, transferred to the laboratory, and sterilized in an autoclave at 121°C for 15 minutes).

### 2.2. Microorganisms and Cultural Conditions

*Streptomyces griseorubens* strain (MMH 9) [17], *Streptomyces rochei* strain (HMM 13) [18], *Streptomyces parvus* strain (8) [19], and *Streptomyces* sp. strain (M12) [20] were isolated from marine sediment samples in the Gulf of Suez and deposited in Genbank with accession numbers KR133201, KR108310, KP675949, and MK388207, respectively, by Dr. Moaz M. Hamed. These marine actinobacterial isolates were maintained on a slant containing starch nitrate agar medium (SNM) with a specific composition per liter: 20 g of starch, 1 g of K_2_HPO_4_, 2 g of KNO_3_, 0.5 g of MgSO_4_, and 18 g of agar. The components were dissolved in 0.5 L of distilled water and 0.5 L of seawater [21]. After autoclaving and cementing, 50 and 20 *μ*g/ml of tetracycline and nystatin were included as antibacterial and antifungal substances, respectively. After seven days, the isolates were incubated at 30-32°C. These isolates were preserved for subsequent examination within the spore suspension of 20% (*v*/*v*) glycerol at -20°C.

### 2.3. Primary Screening of Extracellular *α*-Amylase Enzyme

*α*-Amylase enzyme production was investigated using a starch casein agar plate with an active actinomycetes culture [22]. The ingredients of the medium per 1 L were incubated at 32°C for seven days: 10 g of starch, 2 g of KNO_3_, 1 g of casein, 0.5 g of MgSO_4_, 0.001 g of FeSO_4_, 1 g of K_2_HPO_4_, 0.1 g of CaCO_3_, 18 g of agar, and 50/50 distilled water [23]. The zone of plate hydrolysis after incubation indicated the positive activity of isolates for *α*-amylase production [24].

### 2.4. Enzyme and Protein Assay

*α*-Amylase activity was assayed, following the Miller method, 1951, and modification of the Jain method, 2020. The substrate was prepared by dissolving 1 g starch in 100 ml of phosphate buffer (pH -6.9) with boiling for 5 min at 80 to 85°C. The standard curve for substrate was prepared using different dilutions of starch solutions in the range of 300 mg/l to 3000 mg/l. One hundred microns of starch solution was added to 100 microns of tested crude enzyme. All tubes were mixed and incubated in a water bath for 20 min at 45°C. Four hundred microns of DNS reagent (0.1 g DNSA dissolved in 10 ml of 10 g/l sodium hydroxide, 2 g/l phenol, and 0.2 g/l sodium sulfide) was added into test tubes after incubation with boiling for 5-10 min. After the addition of 2 ml of 40% sodium potassium tartrate, the measuring of absorbance occurred in 575 nm with calculation of amylase concentration using the standard curve [25, 26].

The protein assay was determined by the Lowry method [25]. After using bovine serum albumin as a standard curve for protein with a concentration range of 0.4 mg/l to 4 mg/l, 700 microns of Lowry solutions (NaOH, Na_2_Co_3_, CuSO_4_, and KNaC_4_H_4_O_6_·4H_2_O) was added to 500 microns of the sample. All tubes were mixed and incubated in the dark for 20 min at room temperature. One hundred microns of the diluted Folin reagent (5 ml of 2 N Folin reagent, 6 ml of distilled water) was added and left in the dark for 30 min at room temperature with an absorbance measurement of 750 nm. The protein content was calculated in mg/ml. The specific activity of *α*-amylase was calculated by dividing the enzyme activity into protein concentration [27].

### 2.5. Enhancement of the Production of *α*-Amylase Enzyme

#### 2.5.1. Statistical Design for Optimization of *α*-Amylase Enzyme

The Plackett-Burman experimental design [28] demonstrated the necessity of the medium components for producing *α*-amylase enzymes through selected isolates using starch casein agar media. The Plackett-Burman design matrix had seven independent variables (Table 1) in eight combinations (Table 2). The baseline control was in row No. 9. High (+1) and low (−1) values were evaluated for each component. Each experiment was replicated twice, and the mean of these values was used as a response. Both trials were conducted in triplicate. The main effect of each variable was estimated using the following equation:

(1) $$\begin{array}{c} E_{xi}=\frac{\left(\Sigma M_{i+}-\Sigma M_{i-}\right)}{N}. \end{array}$$

When *E*_*xi*_ is the variable main effect, the *α*-amylase production radiuses for the tests were *M*_*i*+_ and *M*_*i*−_, in which the independent variables were present at high and low concentrations, respectively, and *N* was used to measure the statistical *t*-values of the equal unpaired samples to determine the variable meaning by dividing by two using Microsoft Excel 2019.

#### 2.5.2. Experimental Verification

Verification tests were conducted with double standards using the predicted optimized medium to validate the Plackett-Burman design statistical analysis results. The production of *α*-amylase enzyme was measured by dividing the activity by the protein concentration to determine the specific activity.

#### 2.5.3. Optimization of Culture Conditions Using the Box-Behnken Design

After assessing the relative importance of the discrete variables, the four most important variables were chosen to determine the optimum levels for *α*-amylase enzyme development. The Box-Behnken design (BBD) was used because it is a surface response methodology (Table 3) [29, 30]. This optimization method includes three key steps: coefficient estimation of the mathematical model, response prediction, and model adequacy verification. The four significant variables elucidated using the Plackett-Burman experimental design for the selected best *α*-amylase enzyme produced by the isolate of the *S. rochei* strain (HMM 13) were starch (*X*_1_), K_2_HPO_4_ (*X*_2_), pH (*X*_3_), and temperature (*X*_4_). The center and tall levels of each variable were assigned as −1, 0, and +1, individually. A framework was built for the 27 trials, alongside the normal values for the four variables. The experiments were performed in triplicate, and the mean values were determined for specific activities. The connection between the free factors and response functions was correlated using a second-order polynomial to actuate the optimal point. The condition for the four components was calculated using the formula below:

(2) $$\begin{array}{c} Y=B_{0}+B_{1}X_{1}+B_{2}X_{2}+B_{3}X_{3}+B_{4}X_{4}+B_{12}X_{1}X_{2}+B_{13}X_{1}X_{3}+B_{14}X_{1}X_{4}+B_{23}X_{2}X_{3}+B_{24}X_{2}X_{4}+B_{34}X_{3}X_{4}+B_{11}X_{11}+B_{22}X_{22}+B_{33}X_{33}+B_{44}+X_{44}, \end{array}$$

where *Y* is the anticipated response; *β*_0_ is the show constant; *X*_1_, *X*_2_, *X*_3_, and *X*_4_ are the free factors; *β*_1_, *β*_2_, *β*_3_, and *β*_4_ are the direct coefficients; *β*_12_, *β*_13_, *β*_23_, and *β*_24_ are the cross-product coefficients; and *β*_11_, *β*_22_, *β*_33_, and *β*_44_ are the quadratic coefficients. Microsoft Excel 2019 was used for examination of the experimental data collected using regression analysis.

### 2.6. Statistical Analysis

Numerous straight relapses were made utilizing Microsoft Excel predictions to determine the significance of the *α*-amylase protein (presented in a specific activity) in terms of *t*-value [31], *P* value, and confidence level. The level of significance (*P* value) was resolved using Student's *t*-test. Every single impact *t*-test assesses the probability that finding the observed effect was pure chance. If this is highly unlikely, then the effect is thought to be caused by the variable when it is below the accepted level, such as 5%. The confidence level reflects a percentage of the *P* value. Activities were assessed using the Microsoft Excel solver add-in program. Each response was simultaneously visualized in three-dimensional graphics created using STATISTICA 10.0 software for the four largest independent factors.

#### 2.6.1. Model Verification

Experimentally, the optimal conditions were verified by optimization experiments. The predictions were examined and compared to the basic conditions, near-optimal conditions, and conditions different from the optimum levels of the independent variables.

### 2.7. Purification of *α*-Amylase Enzyme

*α*-Amylase was purified from *S. rochei* HMM 13 using various steps, including precipitation of ammonium sulfate, dialysis, and Sephadex G-50 [32]. All purification steps were completed at 4°C. Overnight, the crude was accelerated by ammonium sulfate with precipitated proteins separated at different saturation percentages (25%, 50%, 75%, and 90%). The precipitated sample was dialyzed overnight against water and barium chloride [33]. The ultimate purification process was performed using Sephadex G-50 with column dimension (2.6 × 20 cm), utilizing a test with 90% immersion precipitation. Sodium dodecyl sulfate-polyacrylamide gel electrophoresis (SDS-PAGE) was used to calculate the molecular weight of the purified enzyme [34].

### 2.8. Characterization of *α*-Amylase Enzyme

The most active peak of the purified enzyme is used for characterization. In this step, nine ml of purified enzyme was used to study pH, temperature, thermal stability, kinetic study, salinity, and metal effect on enzyme activity. The pH (3–10) of purified *S. rochei* HMM 13 *α*-amylases was tested under standard assay conditions. The temperature effect was determined on sterilized *α*-amylase by incubating the *α*-amylase activity at a temperature extending from 30°C to 80°C utilizing phosphate buffer for 4 h and measuring activity as already described [35]. Thermal stability of *α*-amylase was determined when the enzyme was incubated at 55°C, 65°C, and 70°C for 4 h. Enzyme activity was determined at regular time intervals of 20 min. Kinetic *α*-amylase activity parameters were calculated at soluble starch concentrations of 0.1 to 1.2 g/l at a pH of 6 and 65°C for 30 min [36]. Double reciprocal plots obtained the Michaelis-Mentent constant (Km) and maximum reaction rate/velocity [37]. Optimum salinity for *α*-amylase activity was examined by activity at various NaCl concentrations (0.2–2.1 M) in phosphate buffer with preincubation of an enzyme at 65°C and a pH of 6 for 30 min under standard conditions. Metal particles (Co^+2^, Cu^+2^, Mn^+2^, Zn^+2^, Mg^+2^, Fe^+2^, and EDTA) were contributed to distinguish their impact on *α*-amylase activity in 0.05 M phosphate buffer at a pH of 6. A measure of 5 ml of metal ion solution and reagents were combined with refined *S. rochei* HMM 13 *α*-amylases. This mixture was kept under 65°C for 30 min, and the enzyme activity was measured as depicted by [38].

## 3. Results

Marine actinomycete isolates were initially assessed qualitatively for extracellular enzyme development. Preliminary screening showed that *α*-amylase is produced from isolated marine actinomycetes on culture plates. Among the actinomycete isolates, the *Streptomyces rochei* strain HMM 13 was very active on starch agar plates with iodine as a detector (24 mm) when compared to other isolates (Table 4). *S. rochei* HMM 13 isolate was Gram-positive, myceliccoenocytic, and branched. It was catalase and protease positive, reduced nitrate, hydrolyzed starch, Voges Proskauer, indole, and H_2_S production negative. Based on 16S rRNA analysis, this organism was previously identified as *Streptomyces rochei*; the sequence was accessed by GenBank no. KR108310.

### 3.1. Optimization of *α*-Amylase Production from Actinomycete Isolates

**Figure 1:**
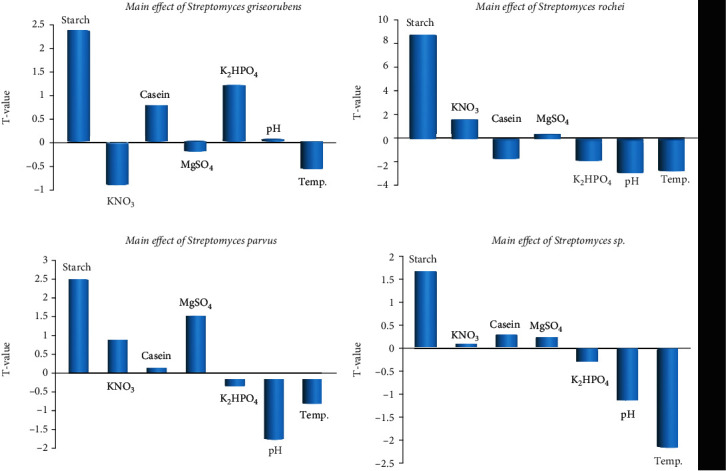
Illustration of fermentation conditions influencing the generation of amylase enzyme by selected actinomycete strains.

The Plackett-Burman design was used to assess the critical impact of utilizing starch casein agar medium components for the generation of *α*-amylase enzyme using *S. griseorubens strain* (MMH 9), *S. rochei* strain (HMM 13), *S. parvus* strain (8), and *Streptomyces* sp. strain (M12). The most significant effect of the four isolates was found for *S. rochei* HMM 13. The *t*-test showed that the most critical independent variables influencing *α*-amylase enzyme development were starch, K_2_HPO_4_, pH, and temperature. Consequently, specific activities were detected (Table 5). The primary effects of the examined factors on the results of the specific activity were estimated, as represented in (Figure 1). Based on these results, the positive (+) level of starch concentration in addition to the negative level (-) of K_2_HPO_4_, pH, and temperature supported production by *S. rochei* isolate. The positive (+) level of starch, casein, and K_2_HPO_4_, in addition to a negative level (-) of KNO_3_, supported production by *S. griseorubens.* However, there was a positive (+) level of starch, KNO_3_, and MgSO_4_ in addition to the negative level of pH corroborative enzyme production by *S. parvus.* Finally, the positive (+) level of starch and negative level (-) of K_2_HPO_4_, pH, and temperature supported enzyme production by *Streptomyces* sp. Additionally, the *t*-value in Table 5 bolsters this perception. This approach has shown that the implemented design is correct. A verification experiment was used to evaluate the basic versus the optimized medium.

#### 3.1.1. Verification Experiment

Verification was achieved by comparing the expected ideal levels of autonomous factors and basic conditions. Cultivation of *S. griseorubens* MMH9, *S. rochei* HMM 13, *S. parvus* (8), and *Streptomyces* sp. M12 in verified medium adjusted to a pH of six for seven days resulted in a 1.7, 9.8, 7.7, and 3.12-fold increment in the specific activity measurement compared to the baseline conditions (Table 6). Among the BBD design, the significant independent variables (starch *X*_1_, K_2_HPO_4_*X*_2_, pH *X*_3_, and temperature *X*_4_) suggested by the Plackett-Burman design were used to investigate the optimum response region for *α*-amylase production by *S. rochei* HMM 13 at three levels (-, 0, and +) in the BBD (Table 7).  Table 8 presents the framework for the variables and the reaction of each trial. To determine the optimal solution, the experimental results were based on a second-order polynomial function (nonlinear optimization algorithm):

*Y* = 4.921 + 0.46*X*_1_ − 0.17*X*_2_ + 1.07*X*_3_ + 0.46*X*_4_–1.51*X*_1_*X*_2_ + 0.55*X*_1_*X*_3_–0.36*X*_1_*X*_4_–0.522*X*_2_*X*_3_–0.33*X*_2_*X*_4_ + 0.94*X*_3_*X*_4_ − 1.15*X*_11_–1.12*X*_22_–0.32*X*_33_–2.57*X*_44_.

**Figure 2:**
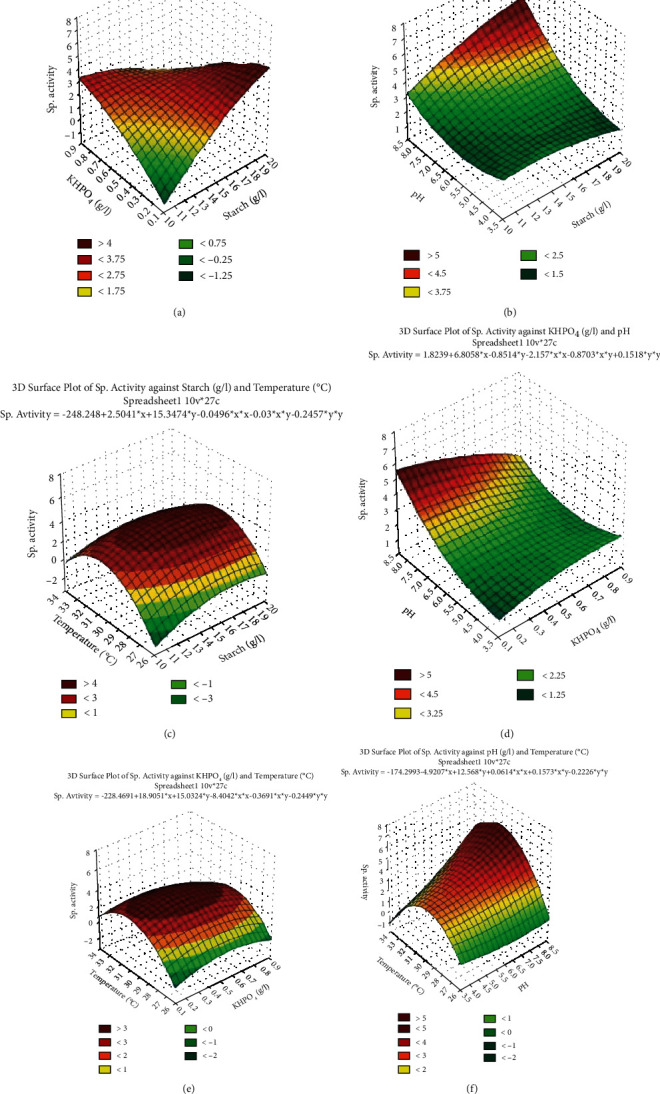
Box-Behnken design: (a) starch, K_2_HPO_4_, and specific activity; (b) starch, pH, and specific activity; (c) starch, temperature, and specific activity; (d) K_2_HPO_4_, pH, and specific activity; (e) K_2_HPO_4_, temperature, and specific activity; (f) pH, temperature, and specific activity.

At the model level, the correlation measures for estimating the regression equation are the multiple correlation coefficient *R* and the determination coefficient *R*^2^. In this experiment, the value of *R*^2^ was 0.824 for *α*-amylase enzyme production by *S. rochei* HMM 13, indicating a substantial degree of correlation between test values and the expected values. The optimum levels of the four factors obtained from the polynomial model were calculated using the Microsoft Excel 2019 solver function and found to be starch: 19 g, K_2_HPO_4_: 0.2 g, pH: 8, and temperature 30.6°C. Also, Figures 2(a)–2(f) demonstrate the simultaneous effects with three-dimensional charts created using STATISTICA 10.0 software for the four most critical independent factors in each response. As shown in the surface plots of the BBD, variations in starch (*X*_1_), K_2_HPO_4_ (*X*_2_), pH (*X*_3_), and temperature (*X*_4_) were effective within the concentration ranges investigated and under the present experimental conditions. These figures suggest that increasing the starch concentration to 19 g/l at a high level of temperature will promote the production of *α*-amylase enzyme. However, a higher level of enzyme production was accomplished with diminished K_2_HPO_4_ concentration.

### 3.2. Verification Experiment

The optimum conditions obtained from the optimization experiment were experimentally verified and compared with the measured data from the model. The positive relationship between anticipated and test values under ideal conditions demonstrates the precision and legitimacy of the model. Consequently, we hypothesized that to produce the highest production of *α*-amylase enzyme by isolate *S. rochei* HMM 13, the medium formula should be kept at 30.6°C for seven days and formulated as follows (g/l): starch 19 g, KNO_3_ 3 g, casein 0.5 g, MgSO_4_ 0.75 g, FeSO_4_ 0.001 g, K_2_HPO_4_ 0.2 g, CaCO_3_ 0.1 g, agar 18 g, and 50/50 DW. An investigative study was conducted to compare the anticipated optimal levels of autonomous factors and fundamental conditions. Development of *S. rochei* HMM 13 within the confirmed medium balanced to pH 8 for seven days resulted in a 12.22-fold increment within the specific activity estimation compared to basal conditions (Table 9).

### 3.3. *α*-Amylase Enzyme Purification and Characterization

**Figure 3:**
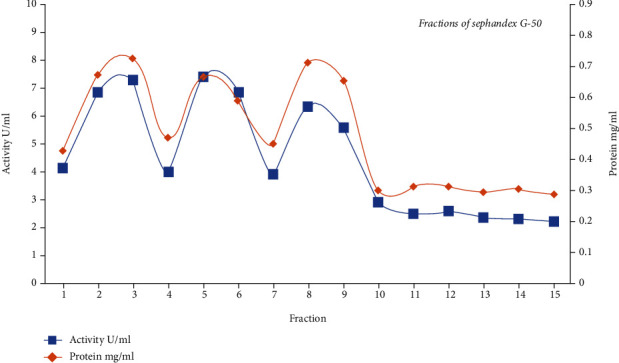
Fractions of amylase production by Sephadex G-50 using *Streptomyces rochei* HMM 13.

**Figure 4:**
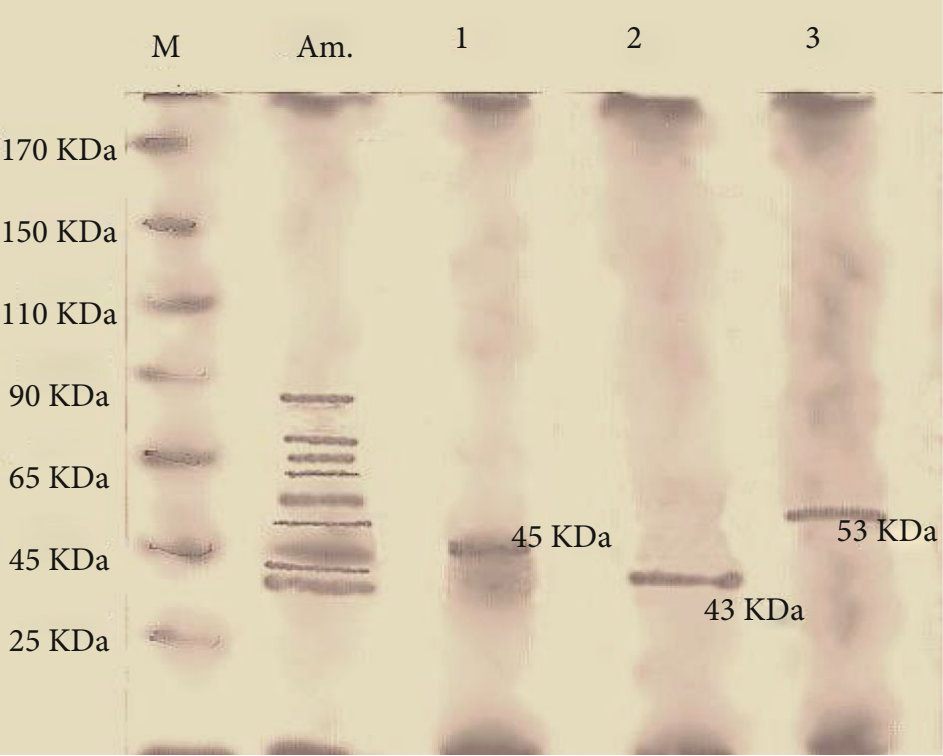
Sodium dodecyl sulfate-polyacrylamide gel electrophoresis for purified amylase enzyme production by *Streptomyces rochei* HMM 13 where Lane 1: marker standard protein with range (25–170 kDa), Lane 2: ammonium sulfate crude, and Lane 3-5: the active peaks of Sephadex G-50.

**Figure 5:**
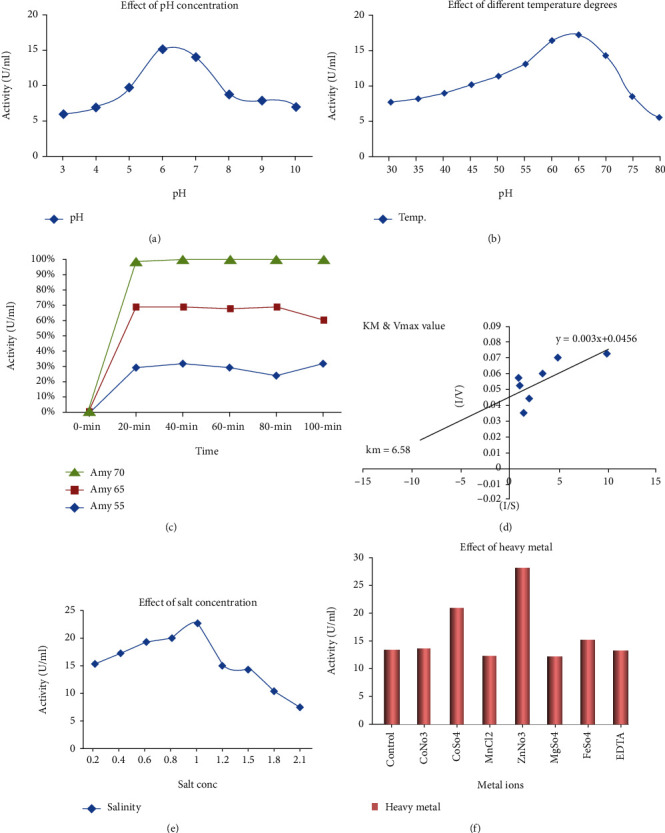
Characterization of purified amylase enzyme production by *Streptomyces rochei* HMM 13: (a) pH effect; (b) temperature effect; (c) thermal stability; (d) Km and Vmax value; (e) effect of salt concentration; (f) metal ion effect.

*α*-Amylase was purified from *S. rochei* HMM 13 using ammonium sulfate, dialysis, and Sephadex G-50. The crude *α*-amylase showed 12.22-fold purification (Figure 3), and the specific activity of *α*-amylase was 7.37 U/mg with that of 64.895 U/ml. At 90%, ammonium sulfate was ideal for the fractionation of *α*-amylase from *S. rochei* HMM 13, and the others showed no significant *α*-amylase activity. The precipitated sample was dialyzed against buffer and water overnight, and the activity of the enzyme was inspected. After purification with Sephadex G-50 chromatography, three curves appeared showing the presence of three different amylase compounds. The first curve includes the first to fourth fractions with an activity 22.442 U/ml and protein 2.305 mg/ml. The second curve includes from the fourth fraction to the sixth fraction with activity 18.396 U/ml and protein 1.736 mg/ml. The third curve appears from the seventh fraction to tenth fraction with activity 4.97 U/ml and protein 1.68 mg/ml. The first peak is the highest activity and protein concentration. Therefore, this peak is used for the characterization step. Dynamic *α*-amylase fractions were stacked onto the SDS-PAGE, and the atomic mass of purified *α*-amylase was 45, 43, and 53 kDa at three peaks within gel filtration chromatography (Figure 4). Purified *α*-amylase was characterized in terms of pH, temperature, thermostability, kinetic activity, salinity, and metal ion. The digestion rate of starch was evaluated with a purified enzyme at different pH values to assess the impact of varying pH on *α*-amylase activity. Enzyme characterization studies revealed stability in a wide pH range (3–10) of purified *α*-amylase with maximum strength and activity at a pH of 6 (Figure 5(a)). In chromatography, there are three peaks, and the most active one is peak number 1 with activity 60% of total specific activity of enzyme which was 10.5 U/mg with an activity of 103.73 U/ml. *α*-Amylase was found to be profoundly dynamic at 65°C (Figure 5(b)). Decontaminated *S. rochei α*-amylase with optimum stability at 70°C was astoundingly steady inside the temperature range 55–70°C. *S. rochei α*-amylase was thermostable up to 70°C and held 52.8% initial activity after 100 min incubation at 70°C. The enzyme maintained its unique action for 40 min at 55°C, 65°C, and 70°C (Figure 5(c)). It was revealed that the *α*-amylase from *S. rochei* is thermally stable. Moreover, the purified enzyme had kinetical characteristics that demonstrate consistent Michaelis-Menten (Km) and Vmax of 6.58 mg/ml and 21.93 mg/ml/min, respectively (Figure 5(d)). The purified enzyme seems to tolerate salinity up to 0.8 M NaCl, with the most elevated movement at 22.71 U/ml (Figure 5(e)). The impact of different metal particles at concentrations of 0.05 M on purified *S. rochei* HMM 13 *α*-amylase activity is shown in (Figure 5(f)). Addition of Cu^+2^, Zn^+2^, and Fe^+2^ for *α*-amylase increased the activity by 152.21%, 207.24%, and 111.89%, respectively. However, Mn^+2^ and Mg^+2^ had a limited impact on enzyme activity by 91.47% and 90.96%, respectively.

## 4. Discussion

The diversity of marine actinomycetes is important in many areas of science and medicine [39]. They are a rich source of bioactive and chemically diverse substances [40]. In this research, actinomycete strains were isolated from the Gulf of Suez just as Ramesh and Mathivanan, who investigate the sediment of Bengal Gulf and confirmed it to be a potential source for aquatic actinomycetes producing industrial enzymes, such as lipase, amylase, cellulase, caseinase, or gelatinase [41]. Amylase is one of the most important industrial enzymes, and it is utilized in many industrial fields in the world. Considering this research, amylase production ability using marine actinomycetes isolated from sediment was investigated as Hwang et al. and Acharyabhatta et al. They produced amylase using *S. avermitilis* and *Streptomyces* BTSS 1001 [36, 42]. Initial screening of studied strains for *α*-amylase production revealed zones of hydrolysis on starch agar plates. The clear zone indicated that *Streptomyces* hydrolyzed starch. Gopinath et al. recently used agar plates to determine the amylase activity of *Penicillium* sp. and *Aspergillus versicolor* [43]. On the contrary, the growth of actinomycetes and the production of enzymes are influenced by the composition of the medium. Therefore, there is a need to urgently change the productivity of enzymes by optimizing bacterial production and use on an industrial scale by physical factors and medium components [44]. Studies to enhance the optimization of marine actinomycete *α*-amylase using statistical instruments were recorded [45]. They applied a Plackett-Burman statistical experimental design to screen eleven medium components based on twelve experimental trials in developing a fermentation medium for *α*-amylase production by *Streptomyces* 4 ALGA, starch, glycerol, maltose, malt extract, glucose, urea, sodium caseinate, yeast extract, peptone, CaCl_2_, and KCl concentrations. The factors that significantly contributed to the maximum *α*-amylase activity were yeast extract and CaCl_2_. The production of *α*-amylase enzymes increased to 23.96 U [45]. Yahya et al. reported that conditions in fungal strain *A. tubingensis* SY1 extracellular amylase production were factually optimized using the Plackett-Burman equation, with the most significant variables of amylase production being peptone, agitation, and MgSO_4_·7H_2_O followed by inoculum size [46]. BBD is a diverse approach that involves answer optimization based on various variable methods, including mathematical and statistical methods [30]. BBD was utilized to optimize the culture conditions for *α*-amylase generation, and a value of 145.32 U/ml of amylase was obtained [47]. Our results agree with Gupta et al., who said that after five days of incubation, the development of *α*-amylase was high at 37°C (108 ± 4.1 U/ml) with a maximum pH of 8.0 (118.1 ± 2.3). The optimal pH and *α*-amylase activity temperature vary from one species to species [14]. The overall production of *α*-amylase in submerged fermentation was 40°C (123.12.1 U/ml). Starch improved the production of *α*-amylase among carbon sources. There was lower *α*-amylase activity in the other carbon sources chosen [44]. The statistical approach of Response Surface Methodology (RSM) using BBD resulted in a doubling of *Streptomyces rochei* biomass yields and indicated the best conditions to establish a cost-efficient approach to enhance *α*-amylase production through variables optimized using BBD. Therefore, RSM can be a useful tool for enzyme development by optimizing the various independent factors involved in the fermentation method using the fewest number of experiments [47].

Purified amylase acts as a natural niche as an industrial biocatalyst. *α*-Amylase is extremely selective, specific, and able to catalyze industrial reactions at low temperature and pressure. Particular *α*-amylase may be specific and selective, but if the contaminant enzymes have opposite (or just different) properties, this may reduce the apparent performance of the prepared biocatalyst. Therefore, obtaining pure enzymes is very important to participate in various industrial reactions, even if these enzymes are in small quantities [48, 49].

Enzyme partial purification of proteins is chromatographically filtered after crude separation by precipitation and membrane separation [50]. The results of partial *α*-amylase purification utilizing *S. rochei* are summarized by ammonium sulfate which is the foremost stable agent for partial filtration [51]. The most common chromatographic columns are sephadex and sepharose, thus separating the active compounds at difference in molecular weight. The *α*-amylase was eluted with a relatively low molecular weight one which was capable of entering the pores easily. The use of an appropriate size of gel particles is the key to the purification process [52]. Therefore, in this research, Sephadex G-50 is the choice for isolation of small molecules of enzymes including *α*-amylase whose molecular weight range is from 35 kDa to 55 kDa. Chromatographic methods for protein purification are the most common methods after crude dialysis and partial precipitation [50]. The efficiencies of purified *α*-amylase immobilization methods are in agreement with other *α*-amylase immobilization studies [53]. The efficiency of the physical adsorption method is similar to that reported by Demirkan et al. for amylase purification using *Bacillus amyloliquefaciens*; the adsorption process of amylase referred to the weak binding of *α*-amylase molecules [54, 55]. Characterization of purified amylase contains pH, temperature, and salt concentration by 6, 65°C, and 0.8 M NaCl, respectively. *α*-Amylases from actinomycetes have a pH optima in the alkaline to a neutral range [44], which differs from our findings. *α*-Amylase was found to be profoundly dynamic at 65°C. Comparable outcomes were found in another study [44], in which the most extreme amylase activity was notable at 60°C. It was revealed that *α*-amylase from *S. rochei* is thermostable. Several previous studies have detailed that amylase from *Streptomyces* sp. MSC702 and *S*. *avermitilis* 5981 were steady at 60°C and 50°C, respectively [36, 37]. The Km and Vmax of the purified enzyme were 6.58 mg/ml and 21.93 *μ*mol/ml/min, respectively, which is close to the Km of *α*-amylase produced by *S. fragilis* DA7-7 by 6.24 mg/ml. Vmax in the current study is higher than Vmax in the study of Nithya and others by 8.36 *μ*mol/ml/min [35]. The 0.8 M of NaCl was the highest degree of salinity for purified *α*-amylase by activity 22.71 U/ml. Previous studies of Anand et al. and Rathore et al. demonstrated that *α*-amylase from *Streptomyces* sp. SNAJSM6 and *N. dassonvillei* KaS11 was steady at 3% and 9% concentrations of NaCl, respectively [56, 57]. Finally, Cu^+2^, Zn^+2^, and Fe^+2^ have positive adding metals for increasing *α*-amylase activity by 152.21%, 207.24%, and 111.89%, respectively, as the result reported by Du et al. [58] and Asgher et al. [59]. The results of the present study indicated the possible testing of the factors that have a positive impact on enzyme production using the PB and BBD.

## 5. Conclusion

The current work used the Plackett-Burman statistical experimental strategy to investigate the major parameters influencing *α*-amylase enzyme production by marine *S. rochei* KR108310 strain HMM 13. To maximize enzyme production, the most effective variables were further adjusted using the Box-Behnken design. The produced enzyme was purified and characterized, being more active at higher temperature and pH, as well as improved by the addition of different metal particles. In general, the characteristics of the pure *α*-amylase enzyme generated by *S. rochei* KR108310 have been investigated, making it a suitable candidate for a wide range of applications.

## Figures and Tables

**Table 1:** Factors of Plackett-Burman design with their high and lower levels.

Variables	Symbol	Ingredients per liter		
		Factor's level	High level (+)	Lower level (-)
Starch	S	10	15	5
KNO_3_	KN	2	3	1
Casein	Ca	1	1.5	0.5
MgSO_4_	Mg	0.5	0.75	0.25
K_2_HPO_4_	K_2_	1	1.5	0.5
pH	pH	7	8	6
Temperature (°C)	T	32	34	30

**Table 2:** The test results for seven cultural variables of the applied design of the Plackett-Burman experimental design.

Trail	S	KN	Ca	Mg	K_2_	pH	T
1	−1	−1	−1	1	1	1	−1
2	1	−1	−1	−1	−1	1	1
3	−1	1	−1	−1	1	−1	1
4	1	1	−1	1	−1	−1	−1
5	−1	−1	1	1	−1	−1	1
6	1	−1	1	−1	1	−1	−1
7	−1	1	1	−1	−1	1	−1
8	1	1	1	1	1	1	1
9	0	0	0	0	0	0	0

S: starch; KN: KNO_3_; Ca: casein; Mg: MgSO_4_; K_2_: K_2_HPO_4_; T: temperature.

**Table 3:** The Box-Behnken experimental design for four factors.

*x*1	*x*2	*x*3	*x*4	*x*1∗*x*2	*x*1∗*x*3	*x*1∗*x*4	*x*2∗*x*3	*x*2∗*x*4	*x*3∗*x*4	*x*1∗*x*1	*x*2∗*x*2	*x*3∗*x*3	*x*4∗*x*4
0	1	0	1	0	0	0	0	1	0	0	1	0	1
1	−1	0	0	−1	0	0	0	0	0	1	1	0	0
0	0	1	−1	0	0	0	0	0	−1	0	0	1	1
1	0	0	1	0	0	1	0	0	0	1	0	0	1
0	1	−1	0	0	0	0	−1	0	0	0	1	1	0
0	0	−1	1	0	0	0	0	0	−1	0	0	1	1
0	0	1	1	0	0	0	0	0	1	0	0	1	1
1	0	1	0	0	1	0	0	0	0	1	0	1	0
−1	0	1	0	0	−1	0	0	0	0	1	0	1	0
0	0	−1	−1	0	0	0	0	0	1	0	0	1	1
0	0	0	0	0	0	0	0	0	0	0	0	0	0
0	1	0	−1	0	0	0	0	−1	0	0	1	0	1
−1	0	0	1	0	0	−1	0	0	0	1	0	0	1
0	1	1	0	0	0	0	1	0	0	0	1	1	0
1	1	0	0	1	0	0	0	0	0	1	1	0	0
0	−1	0	−1	0	0	0	0	1	0	0	1	0	1
1	0	0	−1	0	0	−1	0	0	0	1	0	0	1
−1	0	−1	0	0	1	0	0	0	0	1	0	1	0
−1	−1	0	0	1	0	0	0	0	0	1	1	0	0
−1	0	0	−1	0	0	1	0	0	0	1	0	0	1
0	−1	−1	0	0	0	0	1	0	0	0	1	1	0
0	−1	1	0	0	0	0	−1	0	0	0	1	1	0
1	0	−1	0	0	−1	0	0	0	0	1	0	1	0
0	0	0	0	0	0	0	0	0	0	0	0	0	0
0	−1	0	1	0	0	0	0	−1	0	0	1	0	1
−1	1	0	0	−1	0	0	0	0	0	1	1	0	0
0	0	0	0	0	0	0	0	0	0	0	0	0	0

**Table 4:** Screening of amylase activity with specific activity by using marine actinomycete strains.

	Average of replicate measurement per 100 ml of sample			
Actinomycete strains	α-Amylase activity (U/ml)	Total protein (mg/ml)	Specific activity (U/mg)	Agar plate clear zone (mm)
*Streptomyces griseorubens* KR133201	7.005 ± 0.103	13.7 ± 0.201	0.513	18
*Streptomyces rochei* KR108310	8.16 ± 0.120	14.56 ± 0.214	0.56	24
*Streptomyces parvus* KP675949	6.89 ± 0.104	15.88 ± 0.234	0.434	22
*Streptomyces* sp. MK388207	5.86 ± 0.086	13.22 ± 0.195	0.443	20

**Table 5:** Measurable investigations of the Plackett-Burman exploratory results.

	Main effect and *t* value of all isolates							
	*S. griseorubens*		*S. rochei*		*S. parvus*		*Streptomyces* sp.	
Variable	ME	*t*‐*v*^∗^	ME	*t*‐*v*^∗^	ME	*t*‐*v*^∗^	ME	*t*‐*v*^∗^
Starch	1.17	2.55	1.43	8.44	1.17	2.55	0.88	1.73
KNO_3_	0.42	0.93	0.26	1.57	0.42	0.93	0.05	0.1
Casein	0.08	0.18	-0.27	-1.61	0.081	0.18	0.15	0.3
MgSo_4_	0.72	1.57	0.066	0.39	0.72	1.57	0.13	0.25
K_2_HPO_4_	-0.15	-0.33	-0.32	-1.77	-0.15	-0.33	-0.17	-0.34
pH	-0.8	-1.76	-0.46	-2.75	-0.8	-1.76	-0.61	-1.21
Temp	-0.37	-0.8	-0.43	-2.58	-0.37	-0.8	-1.15	-2.26

^∗^ *t* value significant at the 1% level = 3.70. *t* value significant at the 5% level = 2.446. *t* value significant at the 10% level = 1.94. *t* value significant at the 20% level = 1.372. Standard *t* values are obtained following (Jones, 1994).

**Table 6:** Verification experiment: amylase enzyme production of strains grown in basal versus verified media using the Plackett-Burman design.

	Actinomycete strains	*A* (U/ml)	*P* (mg/ml)	SA (U/mg)	Agar plate (mm)	Purification fold
Basal medium	*S. griseorubens* MMH9	7.004	13.65	0.513	22	1
	*S. rochei* HMM 13	8.16	14.556	0.56	24	1
	*S. parvus*	6.9	15.88	0.434	18	1
	*Streptomyces* sp. M12	5.86	13.22	0.443	20	1

Verified medium	*S. griseorubens* MMH9	8.12	13.69	0.59	27	0.37
	*S. rochei* HMM 13	30.001	10.82	2.77	33	3.97
	*S. parvus*	26.7	11.96	2.23	30	3.34
	*Streptomyces* sp. M12	9.92	10.85	0.91	28	1.06

**Table 7:** The three levels of significant independent variables used in the Box-Behnken factorial experimental design for amylase production by *Streptomyces rochei* HMM 13.

Level	Starch (*X*_1_)	K_2_HPO_4_ (*X*_2_)	pH (*X*_3_)	Temperature (°C) (*X*_4_)
1	19 g	0.8 g	8	33
0	15 g	0.5 g	6	30
-1	11 g	0.2 g	4	27

**Table 8:** Box-Behnken factorial experimental design for amylase production by *Streptomyces rochei* HMM 13.

Trials	Starch (*X*_1_)	K_2_HPO_4_ (*X*_2_)	pH (*X*_3_)	Temp. (°C) (*X*_4_)	*A* (U/ml)	*P* (mg/ml)	SA (U/mg)
1	0	1	0	1	17.8227	10.8877	1.63696
2	1	-1	0	0	14.1576	63.8529	0.22172
3	0	0	1	-1	10.9595	13.2233	0.82880
4	1	0	0	1	13.0436	12.6484	1.03125
5	0	1	-1	0	12.0375	45.4552	0.26482
6	0	0	-1	1	16.6729	12.7562	1.30704
7	0	0	1	1	14.5888	71.8300	0.20310
8	1	0	1	0	14.5528	99.3906	0.14642
9	-1	0	1	0	16.3854	37.3703	0.43846
10	0	0	-1	-1	15.7027	12.9358	1.21388
11	0	0	0	0	18.7210	71.0754	0.26339
12	0	1	0	-1	15.5949	11.3548	1.37341
13	-1	0	0	1	18.6492	41.0354	0.45446
14	0	1	1	0	15.3793	68.8835	0.22326
15	1	1	0	0	19.3319	8.55205	2.26050
16	0	-1	0	-1	13.9779	13.7982	1.01302
17	1	0	0	-1	14.3372	18.1461	0.79009
18	-1	0	-1	0	13.0077	12.7562	1.01971
19	-1	-1	0	0	15.1277	24.8297	0.60926
20	-1	0	0	-1	13.4389	14.1935	0.94683
21	0	-1	-1	0	16.4932	29.3572	0.56181
22	0	-1	1	0	15.5949	71.2910	0.21875
23	1	0	-1	0	13.5108	44.7365	0.30200
24	0	0	0	0	12.4328	68.0930	0.18258
25	0	-1	0	1	11.5704	25.4405	0.4548
26	-1	1	0	0	14.1216	50.7014	0.2785
27	0	0	0	0	11.3189	62.1640	0.1820

**Table 9:** Progression of purification fold for amylase production using *Streptomyces rochei* HMM 13.

	*A* (U/ml)	*P* (mg/ml)	SA (U/mg)	Ap (mm)	Protein recovery	P. fold	Yield%
Basal medium	0.227	0.407	56	24		1	100
Plackett-Burman	30.001	10.82	277	33	2658	3.97	64.47
Box-Behnken	64.895	8.803	737	45	2162	12.22	24.23
Ammonium sulfate (90%)	67.91	7.22	940	—	1773	15.58	18.9
Sephadex G-50							
First peak	22.442	2.305	973		566.34	16.12	19.56
Second peak	14.364	1.261	1138		309.8	18.85	22.87
Third peak	14.966	1.68	890		412.78	14.74	17.88

Note: *A* (U/ml): activity; *P* (mg/ml): protein; SA (U/mg): specific activity; Ap (mm): agar diffusion plate; P. fold: purification fold.

## Data Availability

The data used to support the findings of this study are available from the corresponding author upon request

## Abbreviations

DNS: Dinitrosalicylic acid
BBD: Box-Behnken design
PB: Plackett-Burman
mm: Millimeter
°C: Celsius
l: Liter
SDS-PAGE: Sodium dodecyl sulfate-polyacrylamide gel electrophoresis
EDTA: Ethylenediaminetetraacetic acid
RSM: Response Surface Methodology
SNM: Starch nitrate agar medium
Km: Michaelis-Menten constant
Vmax: Maximal velocity.
